# Disruption of cell adhesion by an antibody targeting the cell-adhesive intermediate (X-dimer) of human P-cadherin

**DOI:** 10.1038/srep39518

**Published:** 2017-01-03

**Authors:** Shota Kudo, Jose M. M. Caaveiro, Satoru Nagatoishi, Takamitsu Miyafusa, Tadashi Matsuura, Yukio Sudou, Kouhei Tsumoto

**Affiliations:** 1 Department of Chemistry & Biotechnology, School of Engineering, The University of Tokyo, Tokyo 108-8639, Japan; 2 Department of Bioengineering, School of Engineering, The University of Tokyo, Tokyo 108-8639, Japan; 3 Biomedical Research Institute, National Institute of Advanced Industrial Science and Technology, Tsukuba, Ibaraki 305-8566, Japan; 4 Perseus Proteomics Inc., Tokyo 153-0041, Japan; 5 Institute of Medical Science, The University of Tokyo, Tokyo 108-8639, Japan

## Abstract

Human P-cadherin is a cell adhesion protein of the family of classical cadherins, the overexpression of which is correlated with poor prognosis in various types of cancer. Antibodies inhibiting cell-cell adhesion mediated by P-cadherin show clear therapeutic effect, although the mechanistic basis explaining their effectiveness is still unclear. Based on structural, physicochemical, and functional analyses, we have elucidated the molecular mechanism of disruption of cell adhesion by antibodies targeting human P-cadherin. Herein we have studied three different antibodies, TSP5, TSP7, and TSP11, each recognizing a different epitope on the surface of the cell-adhesive domain (EC1). Although all these three antibodies recognized human P-cadherin with high affinity, only TSP7 disrupted cell adhesion. Notably, we demonstrated that TSP7 abolishes cell adhesion by disabling the so-called X-dimer (a kinetic adhesive intermediate), in addition to disrupting the strand-swap dimer (the final thermodynamic state). The inhibition of the X-dimer was crucial for the overall inhibitory effect, raising the therapeutic value of a kinetic intermediary not only for preventing, but also for *reversing*, cell adhesion mediated by a member of the classical cadherin family. These findings should help to design more innovative and effective therapeutic solutions targeting human P-cadherin.

P-cadherin is a cell adhesion protein expressed in squamous epithelium^1^,^2^,^3^, but whose overexpression is strongly associated with a poor prognosis in lethal cancers including lung, pancreatic, and breast cancers^4^,^5^,^6^. In particular, overexpression of P-cadherin has been linked to cancer, cell metastatic dissemination, and aberrant growth^6^,^7^. It has been reported that the anti-P-cadherin antibody PF-03732010 blocks cell adhesion, exhibits anti-proliferation activity, and suppresses malignant cell growth *in vivo*^6^,^7^,^8^,^9^, raising the therapeutic value of treatments targeting P-cadherin. Usui *et al*. reported that anti P-cadherin antibodies abolish cell adhesion mediated by P-cadherin, inhibiting cancer cell metastasis and implantation, and inducing anoikis in epithelial ovarian cancer cells^10^. All these results reinforce the suitability of P-cadherin as a promising target in cancer therapy, and establish the concept that disrupting cell adhesion could be a valuable strategy to combat certain types of cancer. However, the molecular mechanism explaining how these antibodies inhibit and/or disrupt cell adhesion mediated by P-cadherin remains unknown. The elucidation of this mechanism may help in the design of novel antibody drugs targeting cancers in which P-cadherin is an oncogenic factor.

P-cadherin is a member of the family of classical cadherins, which is the best-characterized group within the super-family of cell adhesive cadherins^11^,^12^. Structurally, classical cadherins consist of three distinctive regions, namely a five extracellular (EC) domain, a single transmembrane domain, and an intracellular domain^13^. Classical cadherins mediate cell adhesion when the extracellular domains of two cadherin molecules, one from each apposing cell, dimerize in a cadherin-specific fashion^12^,^14^. The basis of homodimerization of classical cadherins has been extensively studied (Fig. 1a)^15^,^16^,^17^,^18^. In particular, the mechanism of cell adhesion by human P-cadherin has also been elucidated at the molecular and cellular levels^19^,^20^,^21^. In these studies we have showed that the so-called strand-swap dimer is the active cell adhesive species, in agreement with what has been reported for other classical cadherins.

Importantly, the formation of the strand-swap dimer is preceded by an intermediate (transient) homodimer termed the X-dimer. The X-dimer is stabilized by intermolecular interactions at the hinge region between EC1 and EC2, and can be stably produced by the addition of a residue, generally a Met residue, prior to the N-terminus of the amino acid sequence^15^,^19^,^22^. The X-dimer thus functions as a thermodynamic and kinetic intermediate platform facilitating the conformational changes from the monomer state to the strand-swap dimer state. Abrogation of the X-dimer by site-directed mutagenesis or by other biochemical means raises the kinetic barrier (activation energy) of association, thereby slowing down the overall rate of strand-swap dimerization. Cell adhesion mediated by P-cadherin is thus facilitated by thermodynamic and kinetic mechanisms that gravitate around the formation of a key intermediate termed the X-dimer.

Antibodies have had a great success as anti-cancer drugs for more than 15 years^23^. They kill tumor cells by directly modifying the function of the antigen (working as agonists or antagonists) or by exposing tumor cells to cytotoxic effector functions such as the antibody-dependent cell-mediated cytotoxicity (ADCC) of the immune response. Engineered antibodies such as antibody-drug conjugates, multi-specific antibodies, and antibody fragments have been vigorously studied and, in some cases, are expected to have an even greater therapeutic effect than intact whole antibodies^24^,^25^,^26^. In the field of cell adhesion, antibodies have been used not only to understand the biological function of various cadherins, but also proposed as experimental treatments in cancers involving E-cadherin and N-cadherin^27^,^28^,^29^,^30^,^31^.

In this study, we have elucidated the mechanism by which an antibody targeting human P-cadherin disrupts cell adhesion. On the basis of our functional and physicochemical analyses, we identified a critical epitope in EC1 at which the antibody TSP7 binds. Surprisingly, cell adhesive dimerization is largely disrupted at the stage of the dimerization intermediate X-dimer, and presumably supported by destabilization of the strand-swap dimer. Crystal structures of the complex between P-cadherin and two different antibodies revealed the structural basis of the antigen-antibody interaction, and why a particular antibody was effective at inhibiting the formation of the X-dimer species. To our knowledge, this is the first report explaining the mechanism of disruption of cell adhesion of a classical cadherin at the molecular level. We anticipate our study will advance the design of novel antibody drugs targeting P-cadherin.

## Results

### Characterization of antibodies TSP5, TSP7, and TSP11

**Figure 1:**
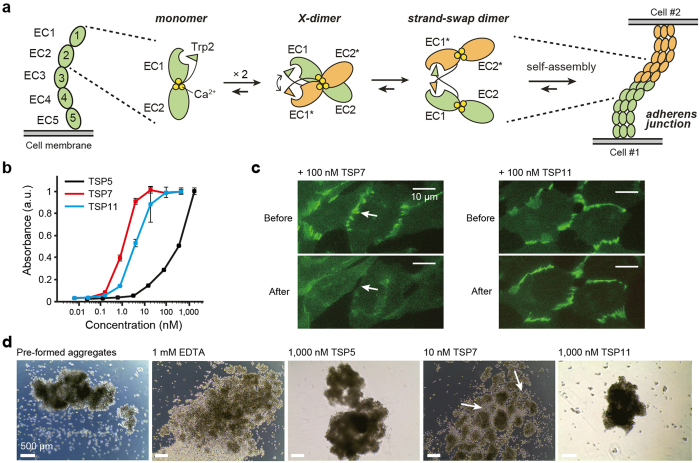
Binding and inhibitory activity of TSP5, TSP7, and TSP11. (**a**) Mechanism of cell-cell adhesion mediated by human P-cadherin. P-cadherin has five extracellular domains (EC1–EC5) of which only EC1–EC2 interact during trans-dimerization. The strand-swap dimer is the stable dimer in the adherens junction and it is formed via the X-dimer intermediate. EC12, colored in green and orange, respectively, represents P-cadherin molecules from different cells. Three Ca^2+^ ions (yellow circles) are bound to the hinge region between EC1 and EC2. (**b**) Binding of antibodies to P-cadherin on cells analyzed by cell ELISA. Plots of TSP5, TSP7, and TSP11 are shown in black, red, and cyan respectively. Each plot represents the mean ± SD. (**c**) Effect of the binding of TSP7 and TSP11 on the localization of P-cadherin-mGFP (mGFP: monomeric GFP). The scale bar indicates 10 μm. The arrows point to a location where disruption of cell adhesion has occurred after the addition of antibody. (**d**) Disruption of cell aggregates by antibodies. Cell aggregates were prepared by incubating cell-samples for 60-min in the presence of 1 mM Ca^2+^, and without antibodies. The pre-formed cell aggregates were then incubated in the presence of 1 mM Ca^2+^, or 1 mM EDTA, or antibodies. TSP5 and TSP11 were added at a higher concentration (1,000 nM) than that of TSP7 (10 nM) to highlight the difference in inhibitory potency. Images were taken after a 60 min incubation. Arrows point at areas of disruption of cell aggregates. Scale bar represents 500 μm.

To elucidate the mechanism of inhibition of cell adhesion by antibodies targeting human P-cadherin, we generated three such antibodies, namely TSP5, TSP7, and TSP11. The three antibodies were expressed and purified in the simplified but functional scFv format with high purity (Supplementary Fig. 1a and b). All antibodies recognized the MEC1 construct of P-cadherin in solution with high affinity (*K*_D_ ≈ 10 nM), and did not cross-react with the highly homologous human E-cadherin (68% identity with human P-cadherin) (Supplementary Fig. 1c–e, and Supplementary Tables 1 and 2). TSP7 and TSP11 also recognized native P-cadherin molecules expressed on the cell surface (EC_50_ ≈ 1–5 nM) (Fig. 1b). The antibody TSP5 was significantly less effective EC_50_ ≈ 300 nM) than the other two antibodies.

Cell imaging was subsequently employed to determine the localization and the effect of P-cadherin when bound to Chinese hamster ovary (CHO) cells. For that purpose, monomeric GFP (mGFP) was genetically linked to the C-terminus of P-cadherin. Characteristic fluorescent puncta were observed at the cell-cell boundary before the addition of the antibodies, suggesting the establishment of typical cell adhesion linkages (Fig. 1c)^21^. However, after the addition of 100 nM TSP7, the fluorescence intensity of the dense puncta decreased sharply within the first 30 min, whereas the puncta were largely unaffected in the presence of TSP11. The results observed with TSP7 are clearly suggestive of a generalized disassembly of the P-cadherin dimers at the cell-cell boundary, thus disrupting cell adhesion.

We examined the effect of the three antibodies in the cell adhesion mediated by P-cadherin by a cell-aggregation assay (Fig. 1d). As a control experiment, we first verified that pre-formed cell aggregates were dissolved in the presence of 1 mM EDTA, a Ca^2+^ chelator capable of subtracting essential Ca^2+^ ions from P-cadherin^21^. Disruption of cell aggregates was observed in the presence of TSP7, but not in the presence of the other two antibodies TSP5 and TSP11. The antibody TSP7 broke up the pre-formed cell aggregates at concentrations as low as 10 nM. The results clearly indicate that of the three antibodies, only TSP7 was effective at disrupting cell adhesion induced by P-cadherin.

### Epitope mapping of TSP5, TSP7, and TSP11

**Figure 2:**
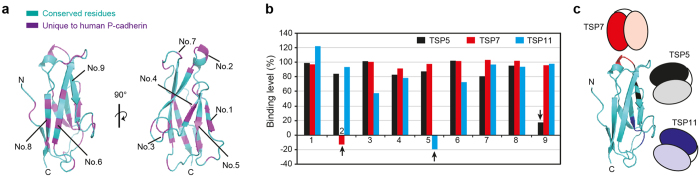
Epitope mapping of TSP5, TSP7, and TSP11. **(a)** Residues unique to human P-cadherin in comparison with human E-cadherin are mapped on the three dimensional structure of EC1. Unique and shared residues are shown in purple and cyan, respectively. The overall sequence identity was 68%. Mutation sites and the description of the individual mutations are summarized in SI Fig. 2a and b. (**b**) Relative binding level for the combination of each mutant to each antibody. The binding level of each antibody to P-cadherin WT is defined as 100% signal. Binding levels of TSP5, TSP7, and TSP11 are shown in black, red, and cyan bars, respectively. **(c)** Location of epitopes of each antibody mapped onto the structure of P-cadherin EC1. Epitopes of TSP5, TSP7, and TSP11 are depicted in black, red, and blue with the corresponding antibodies schematically represented next to them.

Epitope mapping was performed to understand the different inhibitory properties of the antibodies. Nine different sets of mutants of P-cadherin were designed, expressed, and purified for these assays. The different sets of mutants were designed by mutating to Ala residues unique to P-cadherin when compared with E-cadherin (Fig. 2a and Supplementary Fig. 2a,b). Interactions between antibodies and mutants were characterized and determined by surface plasmon resonance (SPR) (Supplementary Fig. 2c). Some of these mutants lost the ability to bind to a certain antibody, but remained strongly attached to the other two antibodies (Fig. 2b). We considered the mutation sites were actual epitopes of the antibody when the binding level was below 20% compared to that of WT cadherin. The location of the epitope of each antibody is schematically depicted in Fig. 2c. It is clear from these results that each antibody recognized a different, and not overlapping, epitope within the surface of EC1. The differences in the inhibitory capacity of each antibody likely reflect a different role of each epitope in the dimerization of P-cadherin (or lack thereof). Among the antibodies examined, the epitope recognized by TSP7 is likely essential for the stability of cell adhesion mediated by P-cadherin.

### Inhibition of the homodimerization of P-cadherin by TSP7

**Figure 3:**
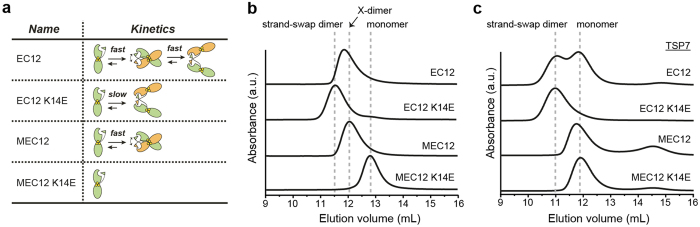
Effect of the binding of TSP7 on the dimerization of P-cadherin. **(a)** Description of the four P-cadherin constructs used in the size exclusion chromatography (SEC) experiments. We note that the X-dimer is a dimerization intermediate, but can be stably prepared when the construct employed is MEC12, containing an additional Met at the N-terminus of EC12. The combination of MEC12 with the point mutation K14E yields a monomeric species. **(b)** SEC profiles of P-cadherin constructs. Each sample at 10 μM was loaded into the SEC column after 60-min incubation at room temperature in the absence of TSP7. Elution volumes of EC12 K14E and MEC12 K14E are indicated by the dotted lines. **(c)** SEC profiles of P-cadherin constructs incubated with TSP7 at 10 μM each, followed by incubation for 60 minutes at room temperature.

To understand how TSP7 disrupts cell adhesion, we examined the effect of the binding of TSP7 to P-cadherin on the dimerization process. Because P-cadherin achieves cell adhesion by forming a strand-swap dimer *via* the kinetic and thermodynamic intermediate state termed X-dimer^21^, we employed several constructs each affecting the dimerization process in a predictable manner (Fig. 3a, Supplementary Table 1)^21^: (i) EC12 samples the three molecular states (monomer, X-dimer, and strand-swap dimer) in a fast exchange of substates, (ii) EC12 K14E is characterized by the slow exchange between monomer and strand-swap dimer and the absence of the intermediate state X-dimer, (iii) MEC12 is distinguished by a fast exchange between the monomer and X-dimer conformations^19^,^21^, and (iv) MEC12 K14E is unable to dimerize, thus remaining as a monomer at all times.

These constructs of P-cadherin were incubated for 60 minutes in the presence or absence of stoichiometric amounts of antibody TSP7, and each separately analyzed by size exclusion chromatography (SEC) (Fig. 3b,c). In the absence of antibody, the strand-swap dimer (EC12 K14E), the X-dimer (MEC12), and the monomer (MEC12 K14E) eluted at increasingly greater volume. The position of the peak depends on the self-affinity and shape of the dimer, as well as the exchange rate between the monomeric and dimeric species (Fig. 3a) and are consistent with previous data^15^,^19^. The importance of the exchange kinetics is manifested by the slightly greater elution volume of EC12 K14E (slow exchange) with respect to EC12 (fast exchange), although we note that in both constructs the strand-swap dimer is the major component.

In the presence of antibody TSP7 the elution volume increased with respect to that in the unbound samples from above, reflecting the higher molecular weight of the P-cadherin/antibody complexes. Specifically, the binding of TSP7 significantly changed the elution profile of two constructs of P-cadherin (Fig. 3c). First, the elution profile of EC12 split into two peaks, one corresponding to the complex of the antibody with the dimer (greater volume) and the second to the monomer (smaller volume). The greater abundance of the peak corresponding to the complex of TSP7 with monomeric P-cadherin with respect to the dimeric complex, suggested that the antibody destabilized the strand-swap dimer, leading to an increase of the concentration of monomer. Second, the complex with MEC12 eluted at a similar position to that of the complex of the monomer (MEC12 K14E). This key observation clearly suggested that the binding of TSP7 is incompatible with the X-dimer. The consequence of this result is that TSP7 would inhibit the fast equilibrium characteristic of EC12. As expected, the complex with the monomer EC12 K14E appears as a single peak. Taken together, these data demonstrate that TSP7 inhibits both the X-dimer and the strand-swap dimer, thus offering an straightforward explanation of the disruption of cell adhesion observed in Fig. 1.

### Structural basis for the inhibition of the X-dimer by TSP7

**Figure 4:**
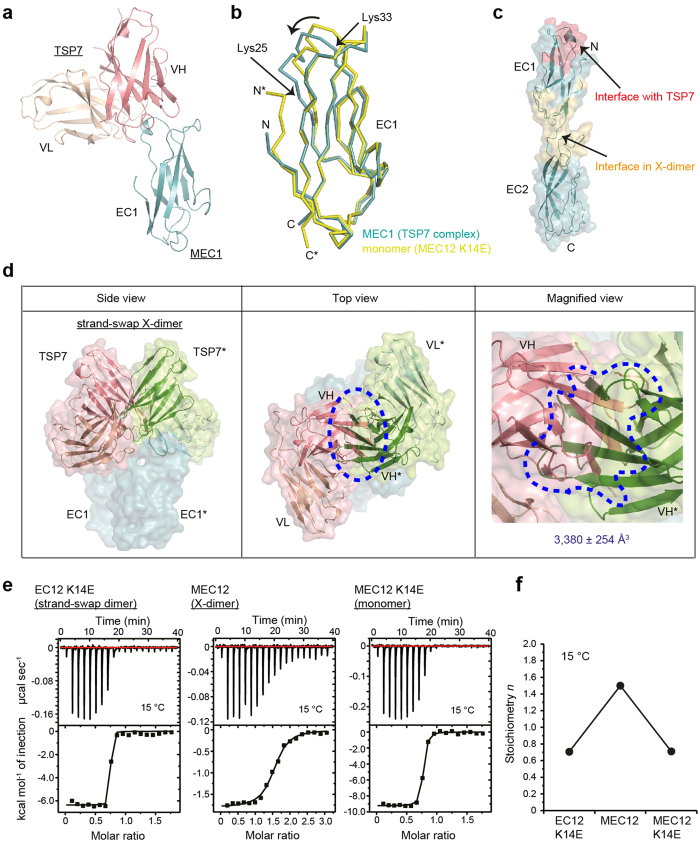
Structural basis for the inhibition of the X-dimerization by TSP7. **(a)** Crystal structure of the complex between TSP7 and MEC1. Heavy and light chains of TSP7 are shown in pink and light brown, respectively. MEC1 is depicted in light teal. **(b)** Comparison of the crystal structures of EC1 in the complex (light teal) and in the monomer (PDB code 4ZMY) (yellow). The arrow points to the region where the largest conformational changes were observed (between Ly25 and Lys33). **(c)** Binding interface of TSP7 (red) and the X-dimer (yellow orange) mapped on the structure of the monomer. **(d)** Superposition of the structures of EC1 in the complex with the antibody and in the X-dimer (strand-swap X-dimer, PDB entry code 4ZMV). The surface of TSP7 is depicted in pink and lime for the complex with TSP7 or with a second molecule of P-cadherin, respectively. The surface of P-cadherin EC1 is colored in light teal. The region where two molecules of TSP7 clash with each other when P-cadherin adopts the X-dimer conformation is highlighted with a dotted line. The overlapping volume was calculated with MSMS^46^. **(e)** Binding isotherm of P-cadherin constructs (EC12 K14E, MEC12, or MEC12 K14E) to TSP7. The experiment was carried out 15 °C. **(f)** Stoichiometry of the reaction determined by ITC.

The crystal structure of the complex between TSP7 and MEC1 was determined at 2.55 Å resolution. Two copies of the complex were found in the asymmetric unit. The root mean square deviation (RMSD) values between the two copies of TSP7, and between the two copies of MEC1 were 0.2 ± 0.31 Å and 0.22 ± 0.16 Å, respectively. The complex displaying the best electron density features was used for the detailed structural analysis (see below). The antibody recognized the loop Gln23–Thr32 of MEC1 (Fig. 4a), consistent with the results of the epitope mapping experiment described earlier (Fig. 2c). Binding of TSP7 to MEC1 did not result in major conformational changes in P-cadherin (RMSD_10-99_ = 0.74 ± 0.44 Å) except at the epitope region (Fig. 4b). A comprehensive list of the non-covalent interactions holding the complex together is provided in Supplementary Fig. 3 and Supplementary Table 3.

At first sight, it was difficult to explain the inhibitory effect of TSP7 since this antibody binds to an epitope far from the interface of the X-dimer (Fig. 4c). To understand the molecular mechanism explaining the inhibitory potency of the X-dimer, we generated a hypothetical dimer by superimposing the coordinates of the cadherin-antibody complex using the X-dimer (strand-swap X-dimer, one class of X-dimer) as a template^21^. We did not observed direct structural features between the TSP7 and P-cadherin molecules that could explain the inhibitory activity of the antibody, such as steric hindrance, or overlap between the binding interface of the X-dimer and the cadherin/antibody complex.

However, when analyzing the relative position of the two TSP7 molecules in the hypothetical dimer, it was clear that a strong overlap between the antibody molecules occurred (Fig. 4d). In particular, the VH domains of each TSP7 molecule notoriously clashed with each other. Indeed the overlapping volume between the two molecules of antibody in this hypothetical complex was exceedingly large (3,380 ± 254 Å^3^). When using another X-dimer as a template to perform the superposition (enc-X-dimer)^21^ the clashes were instead observed between the VH and VL domains, and the total overlapping volume was 1,790 ± 1,200 Å^3^ (Supplementary Fig. 4). In contrast, no structural clashes between the molecules of TSP7 were observed when the strand-swap dimer was employed as a dimeric template. This structural analysis suggests that TSP7 inhibits the X-dimer through steric hindrance between the antibody molecules on two P-cadherin monomers.

This mechanism was validated in solution by isothermal titration calorimetry (ITC). In this experiment, we determined the binding stoichiometry of TSP7 to various constructs of P-cadherin. To minimize the dissociation of the homodimer, the analysis was performed at 15 °C. The results clearly indicate that the stoichiometry increased from a value consistent with a equimolecular binding reaction (*n* = 0.7–0.8) when the partner of the antibody was EC12 K14E (strand-swap dimer) or MEC12 K14E (monomer), to a value double of that (*n* = 1.5) when TSP7 was titrated with MEC12 (Fig. 4e, f and Supplementary Table 4). In other words, only one molecule of TSP7 binds to one molecule of X-dimer, in agreement with the hypothesis elaborated above. Collectively, these crystallographic and thermodynamic analyses have revealed the mechanism by which the presence of the antibody inhibits the formation of the X-dimer in solution: the strongly bound antibody is sterically incompatible with the dimerization of P-cadherin in the intermediate state (X-dimer).

### Structural basis for the inhibition of the strand-swap dimer by TSP7

**Figure 5:**
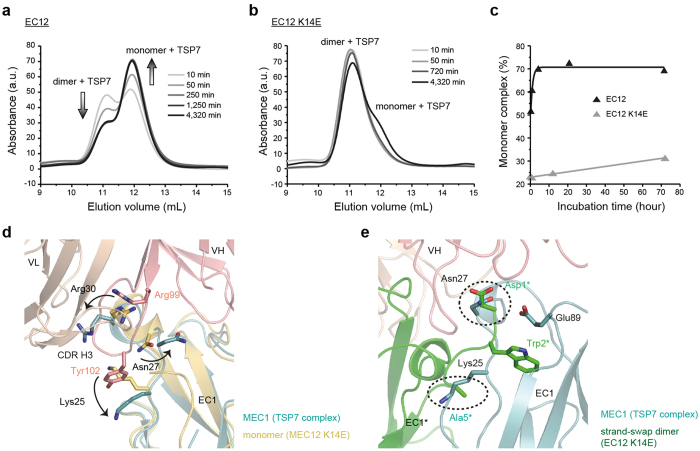
Structural basis for the inhibition of the strand-swap dimerization by TSP7. (**a**) Time-dependent inhibition of the strand-swap dimer of EC12 by TSP7 monitored by SEC. Samples were analyzed 0.2, 1, 4, 21, and 72 hours after incubating the mixed proteins (10 μM) at room temperature. The arrows indicate the increase or decrease on the peak size as the incubation time prior to the SEC experiment increased. **(b)** Analogous experiment with the same antibody but employing the EC12 K14E construct. Samples were analyzed after incubation for 0.2, 1, 4, 21, and 72 hours. **(c)** Time dependence of the intensity of the peak corresponding to the complex between TSP7 and monomeric P-cadherin. The percentage was calculated by using the intensities of the peaks of the dimer and monomer complexes: 100 × I_monomer_/(I_monomer_ + I_dimer_). Black and gray lines corresponds to the data obtained with samples containing EC12 and EC12 K14E, respectively. **(d)** Conformational changes in EC1 induced by the binding of TSP7. Structures in pink, wheat, yellow, and light teal represent the crystal structures of the VH domain of TSP7, the VL domain of TSP7, EC1 in the monomeric form, and EC1 in complex with the antibody, respectively. The arrows point to the conformational changes of three specific residues of P-cadherin: Lys25, Asn27, and Arg30. **(e)** The conformational change induced by the binding of the antibody in the side chains of Lys25 and Asn27 presumably destabilize the strand-swap dimer by direct clashes of these residues with the interface where dimerization occurs. EC1 of the complex was superposed with that of the strand-swap dimer of EC12 (PDB code 4ZML). Only chain B of the strand-swap dimer is shown (dark green). Note that TSP7 recognizes chain A of the strand-swap dimer. The complex with the antibody is shown in the same color as that used in panel (a). Residues potentially clashing in the strand-swap dimer interface are indicated by the dotted circles.

It was found that TSP7 not only disrupted the X-dimer, but also destabilized the strand-swap dimer in the construct EC12, although not in the construct EC12 K14E (Fig. 3c). To understand this complexity, we first examined the stability of the dimer of EC12 and that of EC12 K14E in the presence of the antibody TSP7. Because the rate of monomer-dimer exchange of EC12 and that of EC12 K14E are significantly different from each other^21^, we analyzed the inhibition of strand-swap dimer formation at various times after the samples were mixed (from 10 to 4,320 min). TSP7 steadily disrupted the strand-swap dimer of construct EC12 in a time-dependent manner (Fig. 5a). The amount of monomer reached a plateau (~70%) after four hours. In contrast, for the construct EC12 K14E, the fraction corresponding to the monomer was minor and only discernable after incubation for 72 hours, thus achieving a rate of inhibition much slower than that of the EC12 construct (Fig. 5b,c). From these experiments we concluded that the rate of monomer-dimer exchange was affecting the route by which TSP7 disrupted the strand-swap dimer.

Next, we carefully examined the relevant crystal structures to understand the molecular basis of the disruption of the strand-swap dimer. Based on the crystal structure from above, we noticed that the antibody TSP7 induced a conformational change in a loop of the epitope comprising residues Lys25 to Arg30, which partially blocks the interface of the strand-swap dimer (Supplementary Fig. 5). In particular, the conformation of the side chains of residues Lys25, Asn27, and Arg30 changed under the influence of the complementarity determining region (CDR) H3 loop of TSP7 (Fig. 5d). Superposition of the complex and the strand-swap dimer revealed that the conformation of the side chains of residues Lys25 and Asn27 in the complex with antibody would specifically clash with residues of Ala5* and Asp1* of the second copy of P-cadherin in the swap-strand dimer (Fig. 5e). The crystal structures thus suggests that TSP7 disrupts the strand-swap dimer in an direct fashion, resulting from the conformational changes induced by the antibody in certain residues of P-cadherin in the monomeric state. Because of the faster exchange between monomer and dimer in EC12, TSP7 has greater opportunities to bind and lock the monomer in the construct EC12 than in EC12 K14E. This rationale thus explains why TSP7 has a greater ability to disrupt the strand-swap dimer in the construct EC12 than in the construct EC12 K14E, since the latter mutant lacks the X-dimer intermediate.

### Validation of the inhibitory mechanism at the cellular level

TSP7 disrupted both X-dimer and strand-swap dimer presumably by inactivating the monomeric species of P-cadherin, resulting in P-cadherin/antibody complexes that are unable to dimerize. To validate this mechanism at the cellular level, we employed live cell imaging to analyze the disruption of cell adhesion mediated by P-cadherin. CHO cells expressing human P-cadherin WT (fast monomer-dimer exchange kinetics) or P-cadherin K14E (slow kinetics) were used for this analysis. The intensity of the dense fluorescent puncta at cell-cell boundaries was quantitatively monitored during the experiment (one hour). An illustrative example of dense puncta is shown in Supplementary Fig. 6.

**Figure 6:**
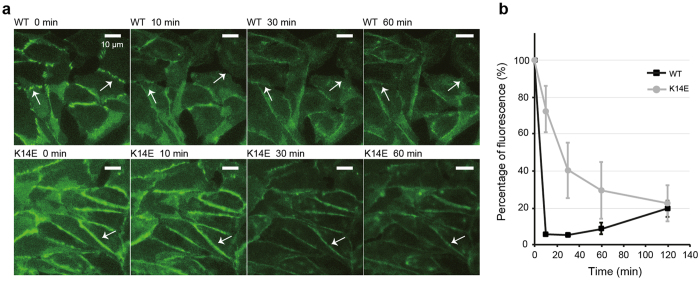
Time-dependent inhibition of the cell adhesion by TSP7. (**a**) Live cell imaging using CHO cells stably expressing human P-cadherin WT-mGFP or K14E-mGFP in the absence and presence of TSP7. Images were obtained with an In Cell Analyzer 2000 instrument (GE Healthcare) with a 60 × 0.7 NA objective lens. Scale bar represents 10 μm. The arrow indicates a typical cell-cell junction region. **(b)** Time-dependent changes in the intensity of the dense fluorescent spots (puncta) at cell-cell boundaries. Dense fluorescence spots were specifically detected and quantified as shown in SI Fig. 6. Initial intensity is defined as 100%. Plots for P-cadherin WT and K14E are shown as black and gray lines, respectively. Plots are the average ± SD.

The presence of TSP7 rapidly diminished the dense fluorescent spots that localized at the cell-cell interface in WT-expressing cells, reducing 90% of the fluorescence puncta within 10 minutes (Fig. 6). In contrast, the dense fluorescent spots disappeared in a significantly slower fashion in cells expressing the construct K14E, requiring 120 minutes to reduce the puncta to 80% of the initial intensity. It is therefore demonstrated that the rate of the monomer-dimer exchange, determined by the residue Lys14 of P-cadherin, governs the kinetics of disruption of TSP7 not only *in vitro* as described above, but also at the cellular level as shown here.

### Rational design of a more potent version of TSP7

We next attempted to design a TSP7 mutant with greater disruption potency. We noticed that although TSP7 inhibits cell adhesion by essentially inactivating the monomeric state, it also interacts with the strand-swap dimer. The binding of two molecules of TSP7 simultaneously to the strand-swap dimer would not only compete with that to the antibody, diminishing its inhibitory potency, but also slows down the dissociation of the dimer because it forces dissociation of the strand-swap dimer by an alternative pathway not involving the X-dimer (Figs 3 and 4). Therefore, we sought to design mutations decreasing the possibility of two molecules of TSP7 to bind simultaneously to the strand-swap dimer, which potentially could boost, even if modestly, the efficacy of our antibody.

**Figure 7:**
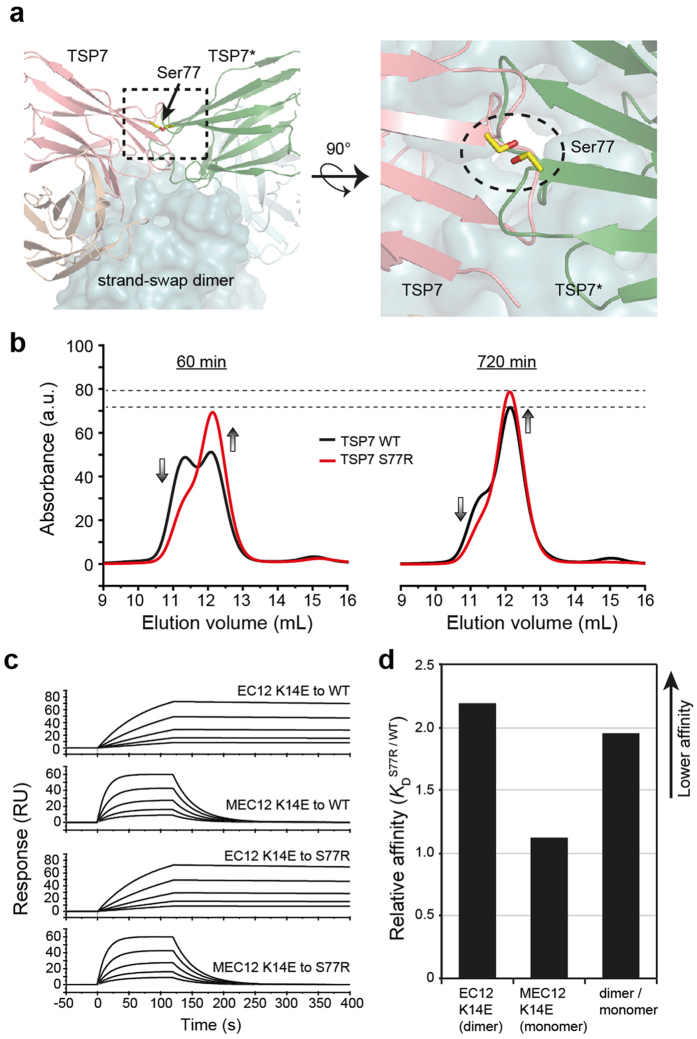
Rational design of a mutation that enhances the inhibitory effect of TSP7. (**a**) Close-up view of the hypothetical conformation of two proximal Ser77 residues of TSP7 facing each other in the strand-swap dimer; EC1 of the complex and the strand-swap dimer are superimposed. Models are represented as in Fig. 4d. (**b**) Comparison of the inhibitory effects of TSP7 and TSP7 S77R on strand-swap dimerization of EC12. Efficiency of the inhibition was analyzed at 60 min and 720 min after mixing. The SEC profiles of TSP7 WT and TSP7 S77R are shown as black and red lines, respectively. **(c)** Binding kinetics between antibodies (TSP7 WT or S77R) and P-cadherin constructs (EC12 K14E or MEC12 K14E) monitored by SPR. **(d)** Affinity for EC12 K14E and MEC12 K14E was compared between TSP7 WT and TSP7 S77R; the *K*_D_ value of TSP7 S77R was divided by that of TSP7 WT.

Based on the crystal structure, we designed the mutation S77R (Fig. 7a). We mutated a small polar residue (Ser) with a bulky and charged residue (Arg) to generate a steric hindrance by the overlap of the two molecules of TSP7 simultaneously bound to the strand-swap dimer. In this mutant, the overlapping volume was estimated to increase from a negative value of −25 Å^3^ (no clashes) to a positive value of 150 Å^3^ (clashes). The mutant S77R of TSP7 was purified to homogeneity in a soluble monomeric form and its inhibitory effect evaluated with SEC. As expected, TSP7 S77R inhibited the dimerization more efficiently than TSP7 WT did at the two incubation times examined, 60 and 720 min (Fig. 7b). From the absorbance of the monomer peak it was estimated that the inhibitory efficiency roughly improved by 10 percentage points (from 70% to 80%).

The basis for the greater inhibitory effect of the mutant TSP7 S77R with respect to the wild-type TSP7 was further examined by comparing the binding affinity of each antibody for the strand-swap dimer and for the monomer (Fig. 7c). As designed, the affinity of TSP7 S77R for the strand-swap dimer was twice as low as that of TSP7 WT, whereas the affinity of each antibody for the monomer remained essentially unchanged (Fig. 7d, Table 1). Note that in relative terms, the *K*_D_ of the antibody for the strand-swap dimer construct was significantly lower than that of the strand-swap dimer most likely because of avidity effect. Collectively, these data demonstrate that the mutation designed on the basis of the mechanism proposed above has produced the desired effects, increasing the potency of an antibody by rational methods, and leading to a more efficient disruption of cell adhesion.

### Molecular recognition of P-cadherin by TSP5 and TSP11

**Figure 8:**
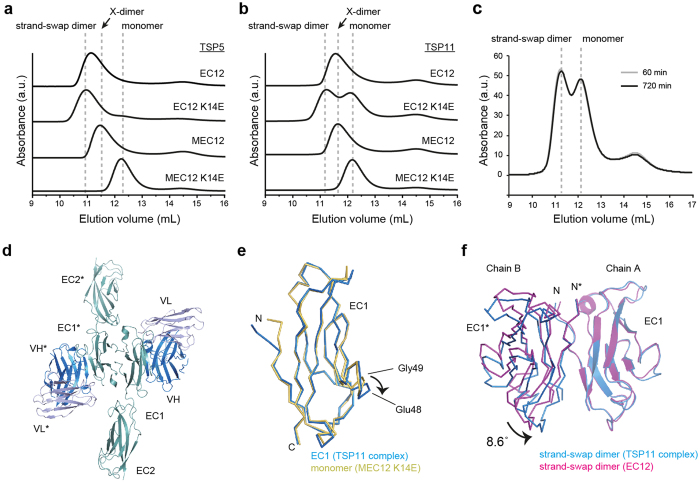
Effect of other antibodies (TSP5 and TSP11) on the dimerization of P-cadherin. (**a)** SEC profiles of P-cadherin constructs (10 μM) in the presence of stoichiometric amounts of TSP5. **(b)** SEC profiles of P-cadherin constructs (10 μM) in the presence of stoichiometric amounts of TSP11. **(c)** Time-dependent SEC analysis of TSP11 bound to P-cadherin EC12 K14E after incubation times of 60 and 720 min. SEC profiles for 60 min and 720 min are shown as gray and black lines, respectively. **(d)** Crystal structure of the TSP11-EC12 complex. Two TSP11 molecules are bound to one strand-swap dimer. The heavy and light chains of TSP11 are shown in blue and light purple, respectively. EC12 is depicted in light teal. **(e)** Superposition of EC1 in the complex with TSP11 (blue) and the monomer (yellow). The arrow points to the conformational changes in Glu48 and Gly49 of P-cadherin. **(f)** Conformational change at the EC1-EC1 interface of the strand-swap dimer in the crystal structure of the complex with TSP11-EC12 (blue). EC1 (chain A) in the complex and EC1 in the strand-swap dimer were superimposed. The arrow highlights the shift of EC1 (chain B).

The mechanism by which the antibodies TSP5 and TSP11 recognize P-cadherin was similarly analyzed. First, the binding of each antibody to various constructs of P-cadherin was monitored by ITC and SEC. In ITC, TSP5 and TSP11 strongly recognized all the P-cadherin constructs examined (*K*_D_ ≈ 10 nM) with a fixed stoichiometry (*n* ≈ 0.7) (Supplementary Fig. 7a–c, Supplementary Table 4). In SEC, TSP5 did not change the elution profile of the P-cadherin constructs (Fig. 8a). However, TSP11 changed the elution profile of EC12 K14E, splitting its elution profile into two peaks (Fig. 8b). The relative intensity of the two peaks did not change over time (Fig. 8c). TSP11 may therefore directly destabilize the strand-swap dimer. Nevertheless, unlike TSP7, neither antibody inhibited X-dimer formation.

In addition, the crystal structure of TSP11 in complex with EC12 was determined at 2.45 Å (Fig. 8d). In the crystal structure, two molecules of antibody are bound to the homodimer species, which is consistent with the stoichiometry observed in the ITC experiment. The list of intermolecular interactions is given in Supplementary Table 5 and illustrated in Supplementary Fig. 8. The binding of the TSP11 antibody to EC12 did not significantly altered the overall structure of EC1 with respect to the unbound form (RMSD_1-100_ = 0.45 ± 0.35 Å) (Fig. 8e), and it did not appreciably changed the combined buried surface are (BSA) value of the interface nor the interactions within the strand-swap dimer (Supplementary Table 6). However, TSP11 slightly altered the conformation of the interface of the dimer (Fig. 8f) by rotating the two EC1 domains by 8.6° with respect to each other. According to this result, TSP11 could directly destabilize the strand-swap dimer by interfering with the hydrogen bond network that stabilize the dimer^21^.

## Discussion

Overexpressed human P-cadherin contributes to the malignancy of cancer cells^6^,^7^, so a promising therapeutic strategy is to develop antibodies that inhibit the cell adhesion of P-cadherin, suppressing malignancy both *in vitro* and *in vivo*^7^,^9^,^10^. However, the mechanism by which antibodies inhibit the cell adhesion of P-cadherin remains unknown. Herein, we elucidated the mechanism explaining the disruption of cell adhesion mediated by P-cadherin by antibody molecules on the basis of structural, physiochemical, and functional analyses.

**Figure 9:**
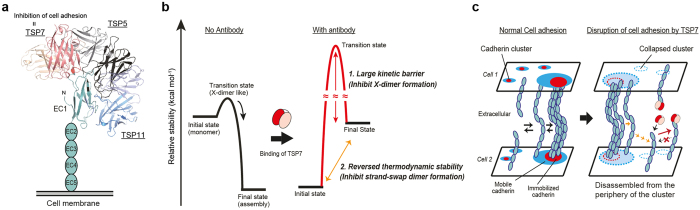
Inhibition of cell adhesion by an antibody targeting P-cadherin. (**a**) Relationship between the epitopes of the three antibodies that recognize EC1 of P-cadherin (TSP5, TSP7, and TSP11). Of the three antibodies, only TSP7 disrupts cell adhesion. The epitope of each antibody is shown in an orientation similar to that in Fig. 2c. The structures of TSP7 and TSP11 were placed through the superposition of the crystal structures. The structure of TSP5 was modeled using CPHmodels^48^; heavy and light chains are colored in black and gray, respectively. The structure of TSP5 was docked manually on the basis of its epitope. **(b)** Energetic description of the inhibition of cell adhesion by TSP7. This antibody inactivates P-cadherin dimerization through two mechanisms. The first is caused by the generation of a “Large kinetic barrier” in the process of homodimerization; this is achieved by inhibiting X-dimer formation. The second is “Reversed thermodynamic stability” between the initial (monomer) and final (strand-swap dimer) states; this is achieved by inhibiting strand-swap dimer formation. TSP7 causes the inactive monomer of P-cadherin to accumulate, resulting in the disruption of cell adhesion. **(c)** Hypothetical molecular mechanism for the disruption of cell adhesion by TSP7. At the adherens junctions, the cadherins form molecular clusters, which consist of concentric mobile (blue) and immobile regions (red). In the mobile region, a fast exchange between monomers and dimers is established. In the immobile region, the cadherin dimers are densely packed and further stabilized. TSP7 may block cell adhesion by disrupting the cadherin clusters at the periphery, as it prevents the homodimerization of P-cadherin by binding to the monomer.

First, we shed light on the importance of the position of the epitope in preventing cell adhesion. Among the three antibodies that recognized EC1 of P-cadherin, only TSP7 blocked cell adhesion. Epitope mapping and crystal structural analysis revealed that these three antibodies recognize different epitopes on EC1. The antibodies showed different inhibitory activity, even though their epitopes appear to be close to each other within the small frame of the EC1 domain (Fig. 9a). In particular, the binding of TSP7 to EC1 abrogated the formation of two different cell-adhesive species, resulting in the disruption of cell adhesion mediated by P-cadherin.

Our findings indicated that the effectiveness of TSP7 is achieved by the simultaneous alteration of the thermodynamic equilibrium (destabilization of strand-swap dimer) and the dimerization kinetic pathway (deactivation of the intermediate species X-dimer) shifting the equilibrium towards the monomeric form (Fig. 9b)^21^. This inhibitory mechanism was further supported after the generation of a mutation in TSP7 (TSP7 S77R), which increased the affinity of the antibody for the monomer species with respect to the strand-swap dimer, increasing the inhibitory potency of the antibody. From our results we have concluded that generation of a stable complex between the antibody and the monomeric species of P-cadherin is essential for the effective disruption of cell adhesion.

Furthermore, we argue that inhibition of the key kinetic intermediate X-dimer contributed most decisively to the disruption of cell adhesion. The antibody TSP11, which binds to the strand-swap dimer but not to the X-dimer, is less effective than TSP7. Disruption of the kinetic regulation by TSP7 is likely effective because it traps the monomeric form of P-cadherin, decreasing the number of free monomer molecules on cells, and accelerating the dissociation of homodimers. We propose a strategy for the design of an effective inhibitor of the cell adhesion mediated by cadherins: the inhibitor should disrupt the kinetic regulation of cell adhesion by impeding the formation of the cell-adhesive X-dimer intermediate (Fig. 9b).

At the cellular level, TSP7 may initiate the disruption of cell adhesion at the periphery of the adherens junctions. Adherens junctions are considered to be the assembly sites of many cadherin clusters^32^,^33^,^34^. Each cluster consists of roughly two regions: an immobilized region and a mobile region (Fig. 9c). In the center of the cluster, the cadherins are densely packed and form the immobile region. Outside the immobilized region the cadherins are loosely assembled, achieving an efficient monomer-dimer equilibrium (mobile region). Given that TSP7 disrupts cell adhesion by the production of a stable monomeric complex, it well could be that TSP7 removes P-cadherin from the mobile region and this continuous uptake from the periphery of the cadherin cluster leads to the disassembly of the core region of the cluster.

Our findings may help in the general development of antibodies that disrupt the cell-adhesive function of cadherins. For example, the design of chimeric antigens may help generate inhibitory antibodies like TSP7. The epitope of TSP7 focuses on residues Asn27 to Asp31, and the residue sequence of this epitope is not well conserved among classical cadherins (Supplementary Fig. 9). Therefore, chimeric cadherin molecules, such as mouse E-cadherin with residues 27 to 31 replaced with those of human P-cadherin, would produce antibodies like TSP7 for various types of classical cadherins via immunization or display technologies, because the mechanism of cell adhesion is common among classical cadherins^15^,^21^. This methodology can also be used to develop drugable antibodies with strong inhibitory function and high stability^27^. In addition to their value as therapeutic drugs, antibodies like TSP7 might also be useful as probes to better understand the mechanism of cell adhesion by classical cadherins.

In conclusion, here we have elucidated the mechanism of disruption of cell adhesion by an antibody targeting human P-cadherin at the molecular and cellular levels. Inhibition of the formation of the cell-adhesive intermediate (X-dimer) led to the accumulation of the inactive P-cadherin monomer–antibody complex, thus blocking cell adhesion. On the basis of these findings, we have proposed a general strategy for the design of antibodies disrupting cell adhesion of classical cadherins. We expect this research will benefit therapeutic approaches against cancers involving P-cadherin, and will deepen our understanding of cell adhesion.

## Methods

All methods were carried out in accordance with relevant guidelines and regulations, and all experimental protocols were approved by the University of Tokyo.

### Production and design of antibody molecules targeting human P-cadherin

TSP5, TSP7, and TSP11 were generated by immunizing mice with whole extracellular domain constructs (EC1–EC5) of human P-cadherin expressed in CHO cells transfected with the pEF4 vector containing the constructs. The DNA sequences of the antibodies were amplified and determined by the rapid amplification of cDNA ends (RACE) method combined with the dideoxy chain-termination method. The single-chain Fv (scFv) format was used to directly assess the effect of antibody binding on the cell adhesion of P-cadherin, as follows: briefly, the VH and VL genes were inserted into pRA2^35^. The VH and VL domains were linked by a flexible (Gly_4_-Ser)_3_ peptide linker. A His_6_ tag was added at the C-terminus of the protein. For expression of the periplasmic region, the pelB leader sequence was placed just before the VH domain. The final construct was pelB-VH-GS-linker-VL-His_6_.i

### Expression and purification of TSP5, TSP7, and TSP11

TSP5, TSP7, and TSP11 were produced in Escherichia *coli*. BL21 (DE3) cells were transformed with the pRA2 vector containing the appropriate antibody gene and were plated on LB plates containing 50 mg mL^−1^ ampicillin at 28 °C overnight. After inoculation into 9 mL of LB medium containing 50 mg mL^−1^ ampicillin, the cells were transferred to 1 L of fresh LB medium supplemented with 50 mg mL^−1^ ampicillin and incubated at 28 °C until the O.D. at 600 nm reached 0.8. Expression of antibodies was induced with 0.5 mM isopropyl β-D-1-thiogalactopyranoside (IPTG) and the cells were cultured for an additional 16 h at 28 °C. The cells were then harvested by centrifugation at 7,000 × *g* for 15 min and resuspended in 50 mL of buffer containing 50 mM Tris, 200 mM NaCl, pH 8.0. Subsequently, the cells were lysed by sonication with an ultrasonic cell-disrupting instrument (Tommy) for 15 min (Output 7, Duty 50). The insoluble fraction containing the antibody was sedimented by centrifugation at 8,000 × *g* for 30 min and sequentially washed three times with 20 mL of Tris buffer (50 mM Tris, 2% Triton X100, pH 8.0), acetone, and deionized water. The proteins were solubilized in binding buffer containing 20 mM Tris, 500 mM NaCl, 5 mM imidazole, 6 M Guanidine-HCl, pH 8.0 at 4 °C overnight. Antibodies were purified in a 1-mL Ni-NTA agarose column (QIAGEN) equilibrated with binding buffer. After the sample was loaded, the column was washed with 10 mL of washing buffer (20 mM Tris, 500 mM NaCl, 10 mM imidazole, 6 M Guanidine-HCl, pH 8.0). Antibodies were eluted with 10 mL of elution buffer (20 mM Tris, 500 mM NaCl, 300 mM imidazole, 6 M Guanidine-HCl, pH 8.0). The antibodies were then refolded by the step-wise dilution method described previously^36^. Finally, the refolded antibodies were purified by size exclusion chromatography (SEC) on a Hiload 26/60 Superdex 75 column equilibrated with buffer containing 10 mM HEPES, 150 mM NaCl, 3 mM CaCl_2_, pH 7.5. The purity of the antibodies was examined by SDS-PAGE and confirmed to be greater than 95%. Antibodies were dialyzed again in SEC buffer before analysis.

### Expression and purification of cadherin constructs

Overexpression of cadherin constructs has been described previously^19^. In brief, each cadherin construct was expressed as a fusion protein with a SUMO tag in Rosetta2 (DE3) cells. Fusion proteins were purified first on Ni-NTA agarose columns followed by cleavage of the SUMO tag with Ulp1^37^. After the SUMO tag and the undigested proteins were removed via the Ni-NTA column, the cadherins were purified by SEC on a Hiload 26/60 Superdex 200 column. Each cadherin construct is shown in Supplementary Table 1. All samples were dialyzed in the SEC buffer before analysis.

### SPR

Interactions between the antibodies and P-cadherin (MEC1) were analyzed in a Biacore T200 instrument (GE Healthcare). The MEC1 construct was immobilized on a CM5 sensor chip by the amine coupling method at a binding level of ~250 RU. Binding of antibodies to MEC1 was determined by increasing the concentration of the analyte (antibodies) from 0.49 nM to 62.5 nM at a flow rate of 30 μM min^−1^ at 25 °C. Measurements were carried out in SEC buffer supplemented with 0.05% (v/v) Tween-20. The association and dissociation times were 120 s and 350 s, respectively. TSP5 and TSP7 were subsequently dissociated from the ligand by regeneration buffer containing 1 M L-Arg (pH 4.4). TSP11 was dissociated from the ligand by regeneration buffer containing 10 mM Gly (pH 2.5). Kinetic parameters (*k*_on_, *k*_off_) and the dissociation constant (*K*_*D*_) were calculated with the BIAevaluation software (GE Healthcare) using a global fitting model.

### Cross-reactivity with human E-cadherin

The cross-reactivity of each antibody with human E-cadherin was analyzed in an iTC200 instrument (GE Healthcare). Antibodies in the sample cell were titrated with human E-cadherin EC12 in the syringe. The concentrations of the antibodies and E-cadherin were 25 μM and 250 μM, respectively. Measurements were performed at 30 °C. After each experiment, the solution remaining in the sample cell was injected into an SEC column to validate the cross-reactivity. Analytical SEC was performed in a Superose 12 10/300 GL column (GE Healthcare) at a flow rate of 0.5 mL min^−1^ at 25 °C. The equilibration buffer (SEC buffer) contained 10 mM HEPES, 150 mM NaCl, and 3 mM CaCl_2_ at pH 7.5.

### Cell ELISA

Binding between antibodies and P-cadherin on cells was analyzed by means of a Cell ELISA. CHO cells expressing human P-cadherin (constructed previously) were used for this analysis^21^. The cells were immobilized on a poly-D-Lys-coated plate (BD Biosciences). Antibodies diluted to various concentrations (0.0064 nM to 500 nM) with Ham’s F12 medium were mounted on each well and incubated for 60 min at room temperature. The cells were then washed three times with saline containing 0.05% Tween-20. Antibodies on cells were detected by 0.75 μg mL^−1^ anti-His_6_-tag antibody conjugated with horseradish peroxidase (HRP) (MBL) in Ham’s F12 medium for 30 min at room temperature. After washing the cells three times with saline containing 0.05% Tween-20 and once with saline without Tween-20, we quantified the binding level with TMB (a colorimetric substrate of HRP). After stopping the reaction with stop buffer, absorbance at 450 nm was measured with a plate reader. Each plot represents the average ± SD.

### Cell imaging

Inhibition of cell adhesion by TSP7 and TSP11 was imaged with an In Cell Analyzer 2000 (GE Healthcare). CHO cells expressing human P-cadherin WT-mGFP (mGFP represents the monomeric form of GFP with the A206K mutation) that was constructed previously were used for the analysis^21^. Cells were seeded onto a 96-well plate (Greiner) and incubated overnight. Images were taken 30 min after the antibodies at the final concentration of 100 nM were loaded at 37 °C with the In Cell Analyzer 2000 using a 60 × 0.7 NA objective lens.

### Cell aggregation assay

Disruption of cell adhesion by TSP7 was analyzed by using the cell aggregation assay described previously in experiments with CHO cells expressing human P-cadherin^21^. Disruption of cell aggregation by TSP7 was analyzed as follows: after Trypsin calcium (TC) treatment, cell aggregates were formed at 37 °C in a rotor spun at 80 rpm for 60 min. Then antibodies at concentrations ranging from 10 nM to 1,000 nM were mixed with the cell aggregates and incubated for 5 min at 37 °C. The cell aggregates were then incubated for a further 60 min at 37 °C in the rotor spun at 80 rpm. After the reaction, images were taken with an Evos XL core using a 4 × 0.13 NA objective lens (Thermo Fisher).

### Epitope mapping (SPR)

Epitope mapping of the three antibodies was performed using a Biacore T200 instrument. To identify the epitope of each antibody, we constructed ten P-cadherin constructs. A description of each mutant is given in SI Fig. 2a and b. Interactions between each antibody with each P-cadherin construct were systematically analyzed by SPR. An anti-His_6_-tag antibody (QIAGEN) was immobilized on a CM5 censor tip through the amine coupling method at a binding level of 11,800 RU. The binding level of P-cadherin mutants was screened as follows: first, 500 nM antibody was loaded and immobilized on the CM5 tip via the anti-His_6_-tag antibody (association: 120 s and dissociation: 200 s). Then, the P-cadherin mutants at a concentration of 1 μM were loaded (association: 120 s and dissociation: 120 s). Finally, both the antibodies and the P-cadherin that remained on the tip were removed for 30 s with regeneration buffer containing 1 M L-Arg at pH 4.4. The binding levels of the P-cadherin mutants were compared with that of P-cadherin WT.

### Analytical SEC

Mixtures of antibodies and P-cadherin constructs were analyzed by use of SEC in a Superose 12 10/300 GL column at a flow rate of 0.5 mL min^−1^ at 25 °C. The equilibration buffer (SEC buffer) contained 10 mM HEPES, 150 mM NaCl, and 3 mM CaCl_2_ at pH 7.5. Equal amounts of antibodies (final 10 μM) and P-cadherin (final 10 μM) were loaded into the column after incubation at room temperature (see figure legends for the incubation times for each experiment). Eluted proteins were detected by monitoring the absorbance at 280 nm.

### Crystallization of complexes of TSP7-MEC1 and TSP11-EC12

Crystallization screens of the complexes of TSP7-MEC1 (5.1 mg mL^−1^) or TSP11-EC12 (8.3 mg mL^−1^) were performed in an Oryx8 instrument (Douglas Instruments) by means of sitting-drop vapor diffusion methods using commercially available kits (Hampton Research) at 20 °C. Promising crystallization conditions were optimized by adjusting the concentrations of the precipitant and salt. Suitable crystals of TSP7-MEC1 were obtained in a solution containing 200 mM potassium phosphate monobasic and 20% (w/v) PEG 3,350. Suitable crystals of TSP11-EC12 were obtained in a solution containing 150 mM calcium chloride and 16% (w/v) PEG 3,350. Crystals of TSP11-MEC1 were cryoprotected with perfluoropolyether oil (Hampton Research). Cryoprotection of the TSP7-EC12 crystals was achieved by briefly immersing them in crystallization solution supplemented with 20% glycerol. Protein crystals were frozen in liquid N_2_ and stored until data collection.

### Data collection and refinement

Diffraction data were collected on the BL5A beamline at the Photon Factory (Tsukuba, Japan) under cryogenic conditions (100 K), and subsequently indexed, integrated, and scaled with MOSFLM and SCALA^38^. The structures of the complexes were determined by the method of molecular replacement using the coordinates of human P-cadherin EC1 or EC12 as models, and a polyalanine model of the anti-carcinoembryonic antigen scFv (PDB entry code 1QOK) with PHASER^39^. Refinement was performed with COOT^40^ and REFMAC5^41^. Structural validation was performed with COOT and PROCHECK^42^. Data collection and refinement statistics are summarized in Table 1.

### Structural analysis

Buried surface area (BSA) and protein-protein H-bonds and salt bridges were obtained with the PISA server^43^. The root mean square deviation (RMSD) (main-chain atoms) was calculated with COMPAR of the CCP4 suite^44^. The rotation angle between EC1 (chain A) and EC1 (chain B) in the strand-swap dimer was calculated with DynDom^45^. Overlapping volumes were evaluated with MSMS of the CHIMERA suite^46^,^47^.

### ITC

Interactions between antibodies and human P-cadherin were examined by means of ITC using an iTC200 instrument (GE healthcare). The sample cell of the calorimeter was filled with antibodies at a concentration range of 15–30 μM. TSP7 was titrated with cadherins at concentrations ranging from 150 to 300 μM. Measurements were performed at 15 °C. Data were analyzed with ORIGIN7 software (GE healthcare) using a one-site binding model.

### Live cell imaging

Inhibition of cell adhesion by TSP7 was quantitatively monitored by using a live cell imaging technique with In Cell Analyzer 2000. Cells expressing human P-cadherin WT-mGFP or K14E-mGFP were seeded onto a 96-well plate (Greiner) and used for the analysis. Images of cells were taken 0, 10, 30, 60, and 120 min after the addition of 100 nM TSP7 in Ham’s F12 medium at 37 °C using a 60 × 0.7 NA objective lens. Data were analyzed with In Cell Developer Toolbox 1.9 (GE Healthcare) using four independent images for each condition. Dense fluorescent dots were specifically detected by the program with the segmentation parameter set as “object” (kernel size = 5; sensitivity = 60) and the intensity of each dense fluorescent dot was calculated. Total intensity was monitored. Values are expressed as the average ± standard error of the mean (SEM).

### Evaluation of the affinity of TSP7 and TSP7 S77R

Differences in affinity between TSP7 and TSP7 S77R for the strand-swap dimer and monomer were assessed by using SPR. scFv (100 nM) was loaded and immobilized for 30 sec onto anti-His_6_-tag antibody immobilized on a CM5 chip. After stabilization of the baseline for 200 s, the strand-swap dimer or monomer construct was loaded as the analyte for 120 s. The dissociation of the analyte was monitored for 300 s; 1.25–20 nM strand-swap dimer and 6.25–100 nM monomer were analyzed. Kinetic parameters (*K*_D_, *k*_on_, *k*_off_) were obtained by using BIAevaluation software with a global fitting model.

## Additional Information

**Accession codes**: The coordinates and structure factors for the structures of the complexes between TSP7 and MEC1 and between TSP11 and EC12 have been deposited in the Protein Data Bank under accession codes 5JYL and 5JYM, respectively.

**How to cite this article**: Kudo, S. *et al*. Disruption of cell adhesion by an antibody targeting the cell-adhesive intermediate (X-dimer) of human P-cadherin. *Sci. Rep.* **7**, 39518; doi: 10.1038/srep39518 (2017).

**Publisher's note:** Springer Nature remains neutral with regard to jurisdictional claims in published maps and institutional affiliations.

## Supplementary Material

Supplementary Information

## Figures and Tables

**Table 1:** Data collection and refinement statistics.

Data Collection	TSP7-MEC1	TSP11-EC12
Space Group	P 1 2_1_ 1	P 2_1_ 2_1_ 2
Unit cell		
*a, b, c*, Å	66.6, 49.4, 111.7	83.4, 199.8, 58.7
α, β, γ, °	90.0, 101.9, 90.0	90.0, 90.0, 90.0
Resolution, Å	54.6 – 2.55	50.6 – 2.45
Wavelength, Å	1.000	1.000
Observations	126,765 (17,049)	245,947 (25,354)
Unique reflections	23,310 (3,274)	36,872 (5,132)
*R*_*merge.*_ (%)^a^	12.5 (67)	8.4 (54)
*R*_*p.i.m.*_ (%)^b^	5.8 (32)	3.5 (26)
Half-set correlation CC_1/2_	0.994 (0.771)	0.997 (0.798)
*I/σ (I)*	10.4 (2.4)	14.5 (2.8)
Multiplicity	5.4 (5.2)	6.7 (4.9)
Completeness (%)	99.1 (96.3)	99.6 (97.6)
**Refinement Statistics**		
Resolution, Å	54.6 – 2.55	50.6 – 2.45
*R*_*work*_*/R*_*free*_, %^c^	21.4*/*27.1	22.3*/*27.0
No. atoms	5,003	6,894
No. protein complexes	2	2
No. residues	645	877
No. waters	84	120
No. other (not solvent)	13	8
Protein B-factor, Å^2^	44.1	52.6
Water B-factor, Å^2^	31.1	32.1
Other B-factor (not solvent), Å^2^	66.9	44.7
Ramachandran Plot		
Preferred Regions, %	93.4	90.1
Allowed Regions, %	6.2	9.6
Outliers (%)	0.4	0.3
RMSD bond, Å	0.008	0.011
RMSD angle, °	1.3	1.5
PDB identification code	5JYL	5JYM

Statistical values given in parenthesis refer to the highest resolution bin.

^a^*R*_*merge*_ = 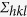 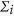
|(*I*(*hkl*)_*i*_ − [*I*(*hkl*)]|/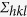 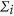 *I*(*hkl*).

^b^*R*_*p.i.m.*_ = 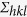 (*I*/(*n*_*hkl*_ − *1*))^1/2^ 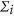
|*I*(*hkl*)_*i*_ − [*I*(*hk*l)]|/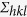 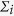 *I*(*hkl*).

^c^*R*_*work*_ = 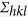 |*F*(*hkl*)_*o*_ − [*F*(*hkl*)_*c*_]|/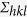 *F*(*hkl*)_*o*_; *R*_*free*_ was calculated as *R*_*work*_, where

*F*(*hkl*)_*o*_ values were taken from 5% of the data not included in the refinement.
