# Supplementary material for: Disruption of cell adhesion by an antibody targeting the cell-adhesive intermediate (X-dimer) of human P-cadherin
Source: Sci Rep. 2017 Jan 3;7:39518. doi: 10.1038/srep39518 (PMC5206748; doi:10.1038/srep39518)
Supplement: Supplementary Information [file srep39518-s1.pdf]

Supplementary Information

Disruption of cell adhesion by an antibody targeting the cell-adhesive intermediate (X-dimer) of human P-cadherin

Shota Kudo[1], Jose M.M. Caaveiro[2], Satoru Nagatoishi[2], Takamitsu Miyafusa[3], Tadashi Matsuura[4], Yukio Sudou[4] and Kouhei Tsumoto[1,2,5,*]

[1]Department of Chemistry & Biotechnology, School of Engineering, The University of Tokyo, Tokyo 108-8639, Japan, [2]Department of Bioengineering, School of Engineering, The University of Tokyo, Tokyo 108-8639, Japan, [3]Biomedical Research Institute, National Institute of Advanced Industrial Science and Technology, Tsukuba, Ibaraki 305-8566, Japan, [4]Perseus Proteomics Inc., Tokyo 153-0041, Japan, [5]Institute of Medical Science, The University of Tokyo, Tokyo 108-8639, Japan.

***** Correspondence should be addressed to Prof. Kouhei Tsumoto, Department of Bioengineering, School of Engineering, The University of Tokyo, Tokyo 108-8639, Japan; Telephone: +813-5449-5316; FAX: +813-64092129; E-mail: tsumoto@bioeng.t.u-tokyo.ac.jp

Supplementary Table 1. Description of the P-cadherin constructs.

Name[a]	Sequence[b]	Strand-swap dimer	X-dimer	Structure	Kinetics[c]
EC12	1-213	Yes	Yes	strand-swap dimer	Fast
EC12 K14E	1-213	Yes	No	strand-swap dimer	Slow
MEC12	1-213	No	Yes	X-dimer	Fast
MEC12 K14E	1-213	No	No	monomer	-
MEC1	1-129[d]	No	No	monomer	-

[a] The letter M refers to the additional Met residue at the N-terminus.
[b] Amino acid numbering is based on the sequence of the mature protein.
[c] Kinetics represents the speed of the equilibrium between the monomer and the dimer.
[d] For the crystallization in complex with TSP7, residues 1-100 of MEC1 were employed.

Supplementary Table 2. Kinetic parameters by SPR.

Antibody	P-cadherin	K_D (nM)	k_{on} (10^5 M^{-1} sec^{-1})	k_{off} (10^{-3} sec^{-1})
TSP5	MEC1	17	18	31
TSP7	MEC1	8.7	30	26
TSP11	MEC1	14	2.8	3.9
TSP7 (WT)	EC12 K14E	0.24	6.2	0.15
TSP7 (WT)	MEC12 K14E	63	4.8	30.0
TSP7 (S77R)	EC12 K14E	0.53	7.3	0.39
TSP7 (S77R)	MEC12 K14E	71	4.5	32.0

Supplementary Table 3. List of non-covalent interactions (H-bonds and salt bridges) in the complex between TSP7 and P-cadherin MEC1.[a]

TSP7	Bond	Distance (Å)	MEC1
Thr30	H-bond	2.9	Lys28
Ala31	H-bond	2.9	Asn27
Asn33	H-bond	2.9	Asp31
His35	H-bond	2.7	Asp31
Asp52	H-bond	2.7	Lys28
Tyr54	H-bond	2.7	Asp90
Arg99	H-bond	2.8	Asp31
Arg99	H-bond	3.0	Asp31
Asp229	H-bond	2.8	Arg30
Arg234	H-bond	2.6	Arg30
His35	Salt bridge	2.7	Asp31
His35	Salt bridge	3.4	Asp31
Asp52	Salt bridge	2.7	Lys28
Asp52	Salt bridge	3.7	Lys28
Arg99	Salt bridge	2.8	Asp31
Arg99	Salt bridge	3.0	Asp31
Arg99	Salt bridge	3.3	Asp31
Arg234	Salt bridge	3.9	Asp31

[a]Individual interactions found in the complex between TSP7 and MEC1 as calculated with the *PISA* program.

Supplementary Table 4. Thermodynamic parameters by ITC.

Antibody	P-cadherin	*n*	ΔG (kcal mol^{-1})	ΔH (kcal mol^{-1})	$-T\Delta S$ (kcal mol^{-1})
TSP5	EC12 K14E	0.78 ± 0.00	-10.5 ± 0.1	-3.4 ± 0.1	-7.1
TSP5	MEC12	0.71 ± 0.00	-10.2 ± 0.1	-7.5 ± 0.1	-2.7
TSP5	MEC12 K14E	0.83 ± 0.00	-9.7 ± 0.1	-5.1 ± 0.1	-4.6
TSP7	EC12 K14E	0.71 ± 0.00	-11.5 ± 0.6	-6.4 ± 0.1	-5.2
TSP7	MEC12	1.5 ± 0.01	-8.5 ± 0.1	-1.8 ± 0.1	-6.6
TSP7	MEC12 K14E	0.74 ± 0.00	-10.4 ± 0.1	-9.2 ± 0.1	-1.1
TSP11	EC12 K14E	0.61 ± 0.00	-9.8 ± 0.1	8.0 ± 0.1	-17.8
TSP11	MEC12	0.71 ± 0.00	-9.3 ± 0.1	7.9 ± 0.1	-17.2
TSP11	MEC12 K14E	0.61 ± 0.00	-10.0 ± 0.1	9.3 ± 0.1	-19.3

Supplementary Table 5. List of non-covalent interactions (H-bonds and salt bridges) in the complex between TSP11 and P-cadherin EC12.[a]

TSP11	Bond	Distance (Å)	EC12
Arg31	H-bond	2.6	Lys64
Arg31	H-bond	2.7	Glu70
Tyr32	H-bond	2.5	Glu48
Trp33	H-bond	2.9	Asn63
Gly104	H-bond	3.2	Ala43
Tyr105	H-bond	3.0	Tyr36
Ser171	H-bond	3.4	Glu56
Ser172	H-bond	3.0	Lys55
Ser172	H-bond	3.4	Glu56
Ser172	H-bond	3.5	Glu56
Glu99	Salt bridge	2.9	Lys64
Glu99	Salt bridge	3.5	Lys64
Arg31	Salt bridge	2.7	Glu70
Arg31	Salt bridge	3.7	Glu70
Arg31	Salt bridge	3.9	Glu70
Arg31	Salt bridge	3.9	Glu70

[a]Individual interactions found in the complex between TSP11 and EC12 as calculated with the *PISA* program.

Supplementary Table 6. Interactions of the strand-swap dimer (construct EC12) in the absence and presence of TSP11.[a]

Structure	Buried surface area (Å^2)	Number of H-bonds	Number of salt bridges
Complex	820	10	4
EC12	844	10	4

[a]Buried surface area, number of H-bonds, and number of salt bridges in strand-swap dimers for the complex of TSP11-EC12, and EC12 as calculated with the *PISA* program.

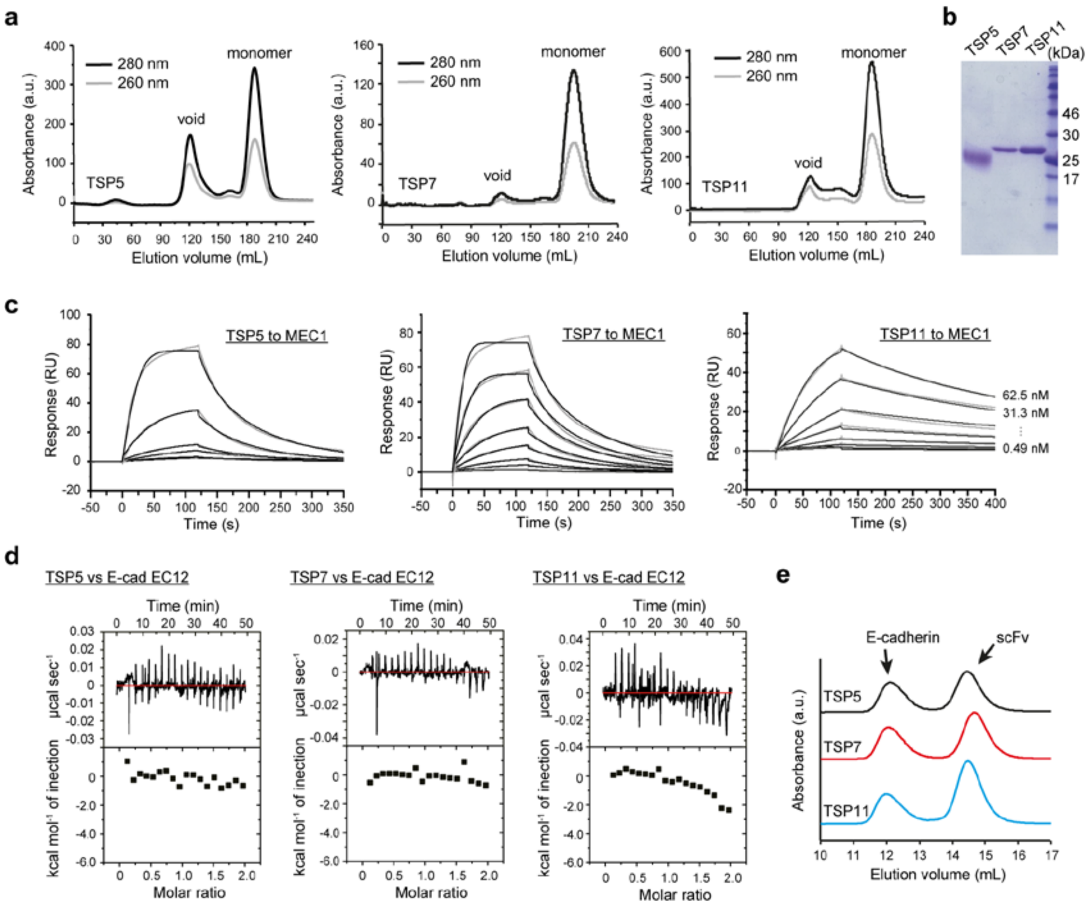

Supplementary Figure 1. Purification and binding activity of TSP5, TSP7, and TSP11. (a) SEC profile of each scFv in the final purification. Absorbance at 280 nm and 260 nm is shown as black and gray lines, respectively. Monomer fractions were collected for the analysis. **(b)** Purity of each antibody evaluated by SDS-PAGE. Molecular weight markers are indicated. **(c)** Sensorgrams of the interactions of TSP5, TSP7, and TSP11 with MEC1. MEC1 was immobilized on a CM5 chip and its interactions were analyzed by changing the concentration of scFv (0.49 – 62.5 nM). **(d)** Cross-reactivity of each antibody with human E-cadherin EC12 analyzed by ITC. Human E-cadherin has high sequence identity (69%) with human P-cadherin. **(e)** Cross-reactivity of each antibody with human E-cadherin analyzed by SEC using ITC samples. E-cadherin EC12 and scFv were eluted separately. Black, red, and cyan lines indicate the elution profiles of the ITC samples of TSP5, TSP7, and TSP11, respectively.

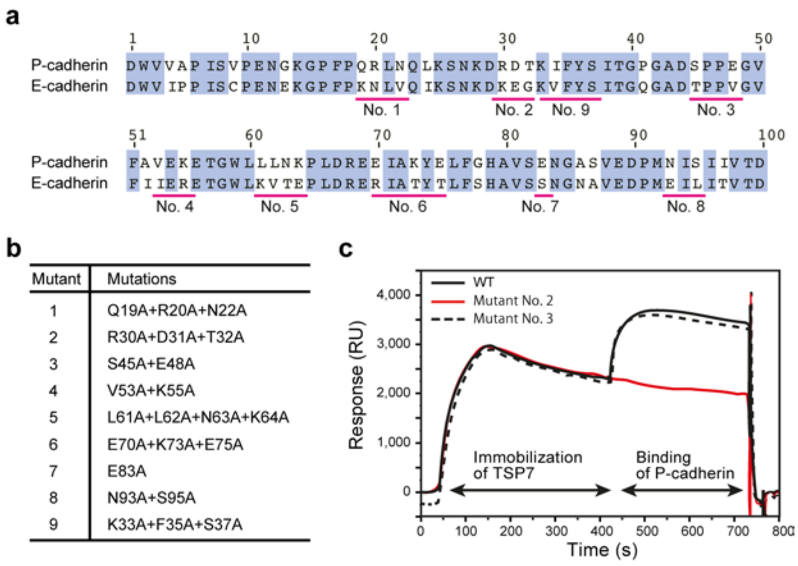

Supplementary Figure 2. Epitope mapping of TSP5, TSP7, and TSP11. (a) Alignment of the amino acid sequences of human P-cadherin and E-cadherin. Identical amino acid residues are shaded blue. Mutated regions are indicated by solid magenta lines. **(b)** List of the nine sets of mutants of P-cadherin EC12 used for epitope mapping and a description of each one. **(c)** Representative sensorgrams during epitope mapping. Antibodies and P-cadherin molecules were sequentially loaded onto anti-His$_6$-tag antibodies covalently immobilized on a CM5 chip. Black solid, red solid, and black dashed lines represent the binding responses of P-cadherin WT, mutant No. 2, and mutant No. 3, respectively, to a surface decorated with TSP7.

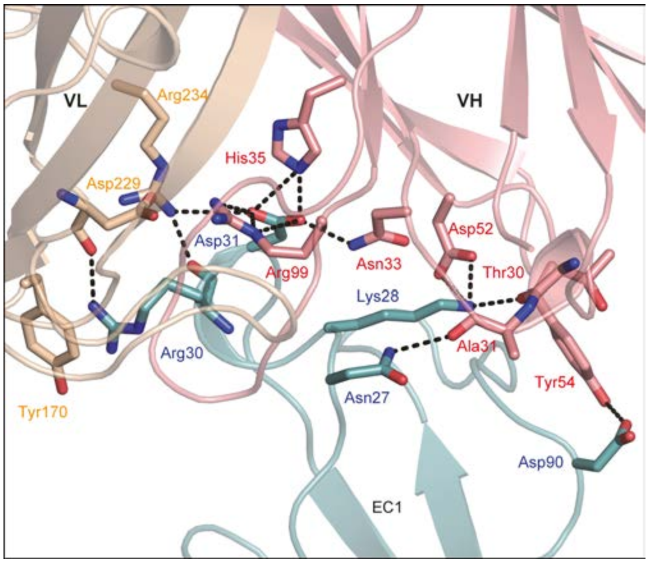

Supplementary Figure 3. Contact interface between TSP7 and MEC1. Hydrogen bonds and salt bridges were identified by using the PISA program. The side chains of the residues that contribute to the interactions are depicted in light teal (MEC1), salmon (heavy chain of TSP7), and orange (light chain of TSP7). The complete list of non-covalent interactions is given in Supplementary Table 3.

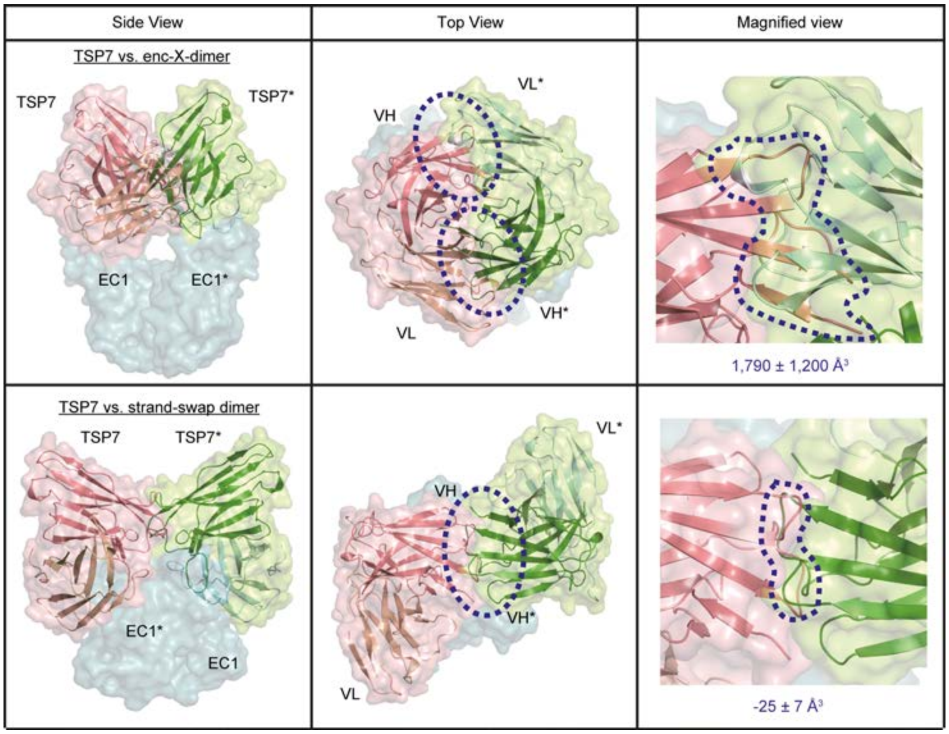

Supplementary Figure 4. Hypothetical clash between two molecules of TSP7 in the enc-X-dimer and in the strand-swap dimer. Superposition of EC1 in the complex with the antibody and the enc-X-dimer or the strand-swap dimer. The surface of TSP7 in one complex and that in a second (hypothetical) complex is depicted in pink and lime, respectively. The surface of EC1 of P-cadherin is shown in light teal. The proximal regions of two TSP7 molecules are indicated by dotted circles. The overlapping volume was calculated with the MSMS program of the CHIMERA suite.

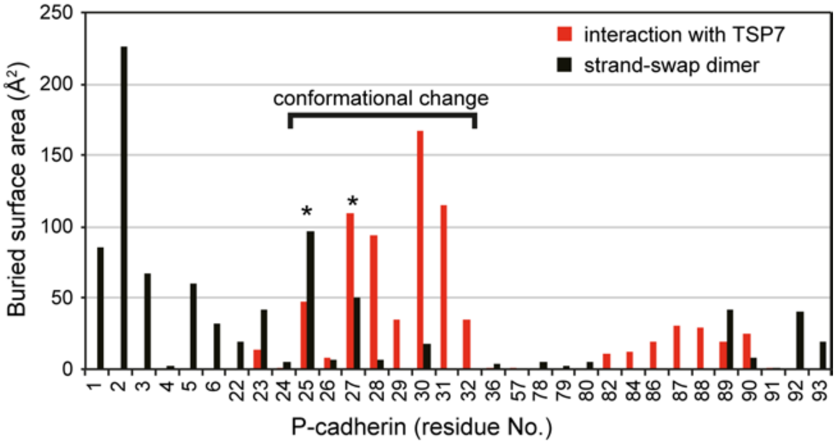

Supplementary Figure 5. Contact interface in the strand-swap dimer of P-cadherin, and in the complex between P-cadherin and TSP7. Buried surface area (BSA) values of each residue involved in the interaction with TSP7 (red) or in strand-swap dimerization (black) are shown. The stars indicate residues that contributed to both interactions above BSA values of 40 Å^2.

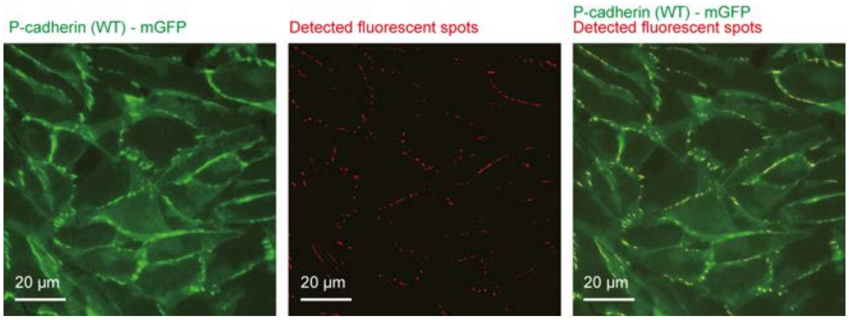

Supplementary Figure 6. Specific detection of dense fluorescent spots at cell-cell junctions. Cell adhesion was quantified by evaluating the intensity of the dense fluorescent spots that localized at the boundary of cells. Dense fluorescent dots were specifically detected in an In Cell Analyzer 2000 instrument. The fluorescence pattern of P-cadherin (WT)-mGFP (green), fluorescent spots detected by the program (red), and the merged images are shown as a representative experiment. The images were taken without antibodies.

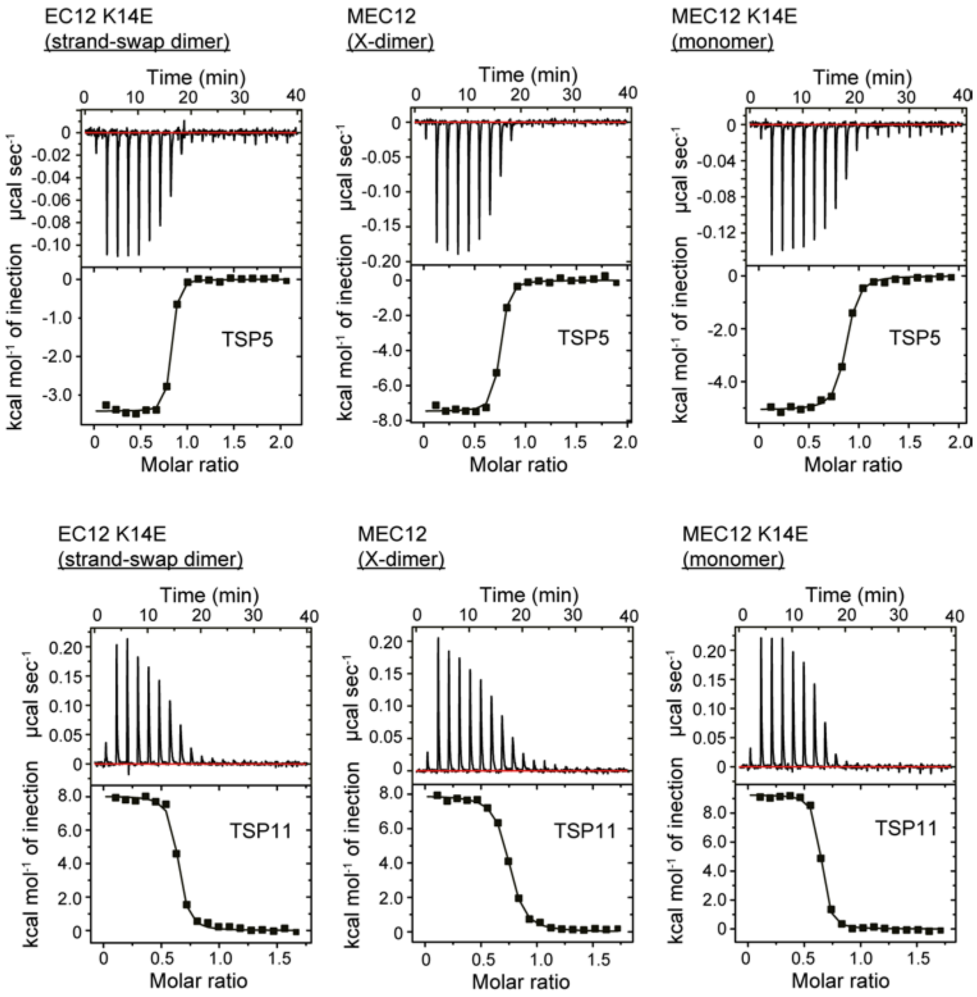

Supplementary Figure 7. ITC profile of TSP5 and TSP11. Binding isotherms of antibodies (TSP5 and TSP11) and P-cadherin constructs (EC12 K14E, MEC12, and MEC12 K14E) measured by ITC at 15 °C. The thermodynamic parameters of each interaction are summarized in Supplementary Table 4. Note that the stoichiometry of each interaction was around 0.7 for interactions between antibodies (TSP5 and TSP11) and P-cadherin constructs.

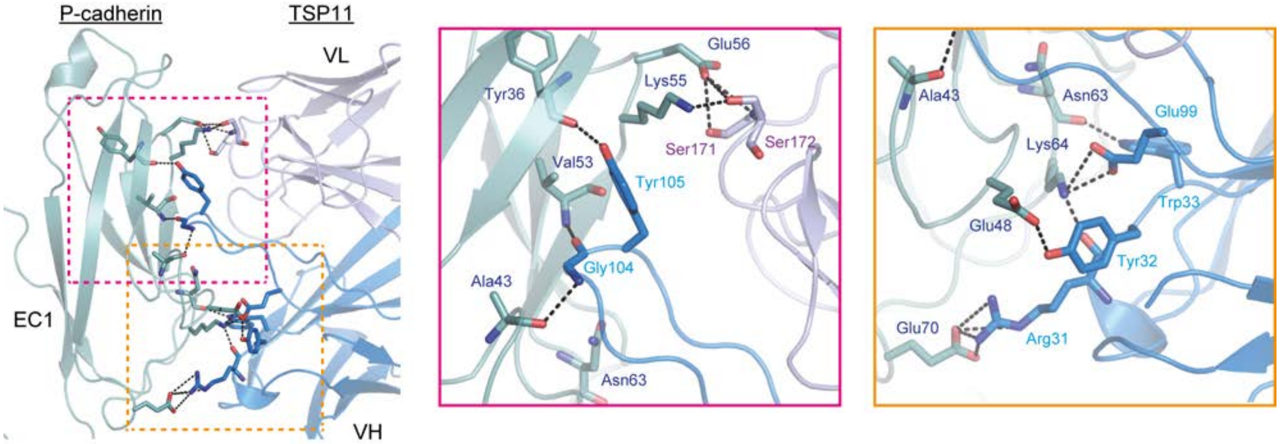

Supplementary Figure 8. Interactions in the crystal structure of the complex between TSP11 and EC12. Hydrogen bonds and salt bridges were identified by using the PISA program. Residues contributing to the interactions are shown in light teal (EC12), marine (heavy chain of TSP11), and light purple (light chain of TSP11). Individual interactions are summarized in Supplementary Table 5. Interactions are shown in two separate areas (pink and yellow boxes) because the epitopes recognized by TSP11 comprise two separate regions of P-cadherin.

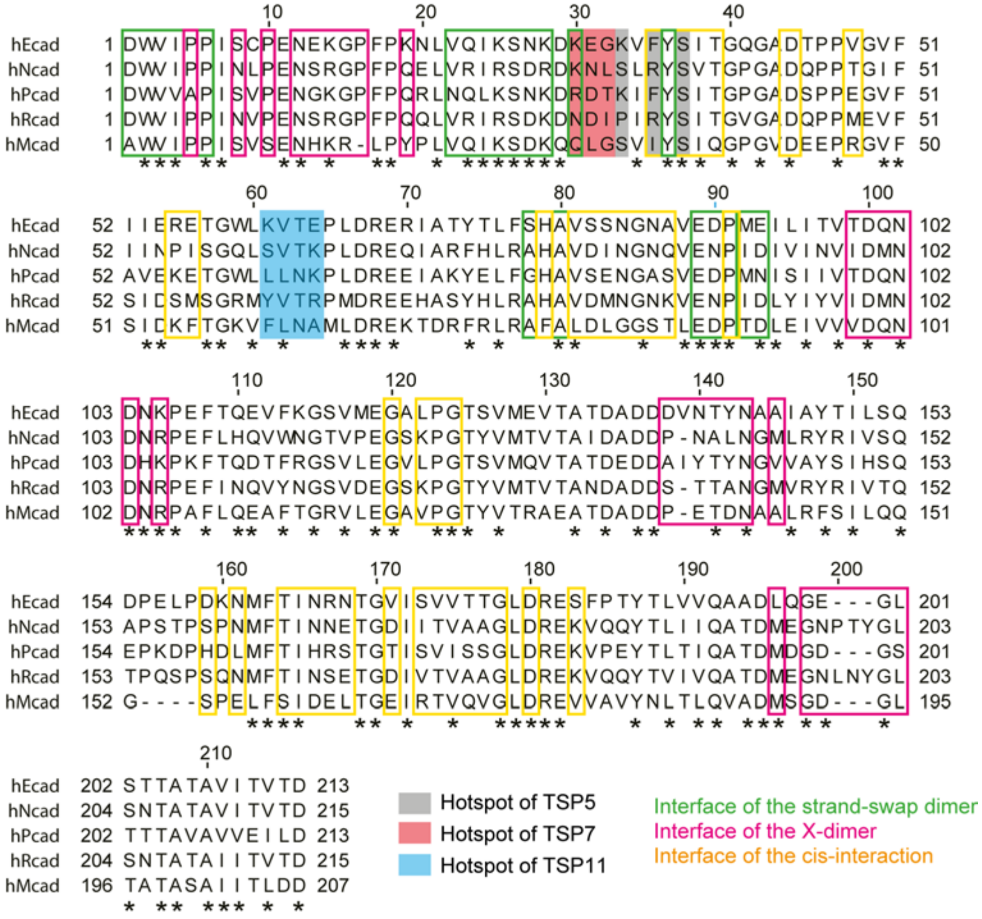

Supplementary Figure 9. Sequence alignment of classical cadherins (type I) and the epitopes of three antibodies (TSP5, TSP7, and TSP11). Residues involved in the formation of the strand-swap dimer, the X-dimer, and the *cis*-interaction are indicated by green, magenta, and yellow boxes, respectively. Residues involved in the interaction with TSP5, TSP7, and TSP11, identified by epitope mapping (Figure 2), are colored in gray, red, and cyan, respectively.
